# Quinoline-2-carbaldehyde

**DOI:** 10.1107/S1600536811035653

**Published:** 2011-09-14

**Authors:** William M. Motswainyana, Martin O. Onani

**Affiliations:** a University of the Western Cape, Cape Town, Bellville 7535, South Africa

## Abstract

The title compound, C_10_H_7_NO, crystallizes with two almost planar mol­ecules (*A* and *B*) in the asymmetric unit (r.m.s. deviations = 0.018 and 0.020 Å). In the crystal, the *A* mol­ecules are linked by weak C—H⋯O inter­actions, thereby generating *C*(9) [001] chains. The *B* mol­ecules do not exhibit any directional bonding inter­actions.

## Related literature

For the synthesis of the title compound, see: Cooper & Cohen (1932 ▶). For its use in the synthesis of Schiff base ligands and imino-quinolyl-based transition metal complexes, see: Amandola & Mangano (2003 ▶); Prema & Wiznycia (2007 ▶); Ramos Silva *et al.* (2007 ▶); Ardizzoia *et al.* (2009 ▶). For its catalytic properties, see: Zhou *et al.* (2008 ▶). 
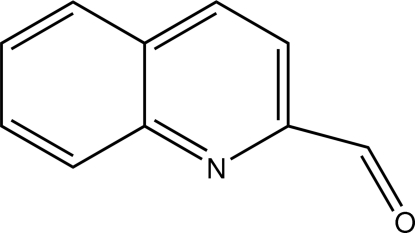


## Experimental

### 

#### Crystal data

C_10_H_7_NO
*M* *_r_* = 157.17
Monoclinic, 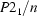

*a* = 7.0639 (7) Å
*b* = 21.564 (2) Å
*c* = 10.698 (1) Å
β = 107.884 (2)°
*V* = 1550.9 (3) Å^3^
*Z* = 8
Mo *K*α radiation
μ = 0.09 mm^−1^
*T* = 173 K
0.16 × 0.09 × 0.06 mm

#### Data collection

Bruker Kappa DUO APEXII diffractometer
17618 measured reflections
3887 independent reflections
2379 reflections with *I* > 2σ(*I*)
*R*_int_ = 0.055

#### Refinement

*R*[*F* ^2^ > 2σ(*F* ^2^)] = 0.045
*wR*(*F* ^2^) = 0.117
*S* = 1.00
3887 reflections
217 parameters
H-atom parameters constrained
Δρ_max_ = 0.20 e Å^−3^
Δρ_min_ = −0.23 e Å^−3^

### 

Data collection: *APEX2* (Bruker, 2006 ▶); cell refinement: *SAINT* (Bruker, 2006 ▶); data reduction: *SAINT*; program(s) used to solve structure: *SHELXS97* (Sheldrick, 2008 ▶); program(s) used to refine structure: *SHELXL97* (Sheldrick, 2008 ▶); molecular graphics: *X-SEED* (Barbour, 2001 ▶); Atwood & Barbour, 2003 ▶); software used to prepare material for publication: *SHELXL97*.

## Supplementary Material

Crystal structure: contains datablock(s) I, global. DOI: 10.1107/S1600536811035653/hb6393sup1.cif

Structure factors: contains datablock(s) I. DOI: 10.1107/S1600536811035653/hb6393Isup2.hkl

Supplementary material file. DOI: 10.1107/S1600536811035653/hb6393Isup3.cml

Additional supplementary materials:  crystallographic information; 3D view; checkCIF report

## Figures and Tables

**Table 1:** Hydrogen-bond geometry (Å, °)

*D*—H⋯*A*	*D*—H	H⋯*A*	*D*⋯*A*	*D*—H⋯*A*
C4*A*—H4*A*⋯O1*A*^i^	0.95	2.53	3.424 (2)	158

Symmetry code: (i) 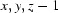.

**Fig. 1.**
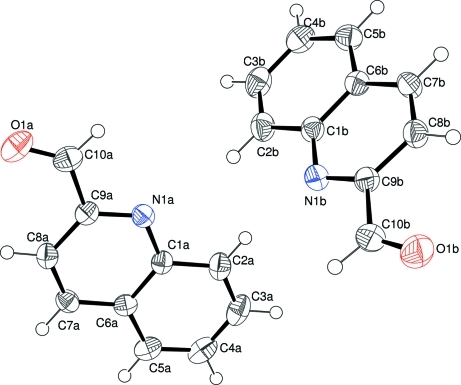
Molecular structure of the title compound showing displacement ellipsoids with probability level of 50%.

**Fig. 2.**
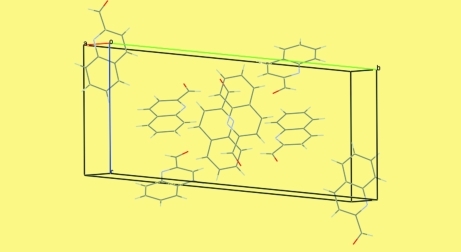
Crystal packing of the title compound.

**Fig. 3.**
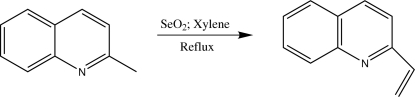
The formation of the title compound.

## supplementary crystallographic information

### Comment

As part of our investigation of bimetallic complexes as catalysts for C—C coupling reactions, we attempted to synthesize palladium (II) complexes of a bis(imino-quinolyl) ligand. The binucleating ligand brings two metal centers into closer proximity and the resultant bimetallic complex possesses unique reactivity patterns and unusual catalytic properties (Zhou *et al.* 2008). In an attempt to prepare a bis(imino-quinolyl) palladium (II) complex, the title compound, (I), waas indavertantly obtained (Fig. 1).   Dimensions are available in the archived CIF.

### Experimental

Single crystals of 2-quinolinecarboxaldehyde were obtained as a result of the decomposition of bis(imino-quinolyl) chloromethyl palladium (II) complex. The bis-palladium (II) complex was prepared from the reaction of a bis(imino-quinolyl) ligand with 2 equimolar PdClMe(cod) in CH_2_Cl_2_. Orange needles of the title compound were grown by slow diffusion of hexane into the CH_2_Cl_2_ solution of the complex.

### Refinement

All non-hydrogen atoms were refined anisotropically. All hydrogen atoms were placed at geometrically calculated positions with d(C—H) = 0.95 Å and refined as riding on their parent atoms with *U*_iso_ (H) = 1.2 *U*_eq_ (C). The structure was successfully refined to *R* factor of 0.0451.

### Crystal data

**Table:**

C_10_H_7_NO	*F*(000) = 656
*M**_r_* = 157.17	*D*_x_ = 1.346 Mg m^−^^3^
Monoclinic, *P*2_1_/*n*	Mo *K*α radiation, λ = 0.71073 Å
Hall symbol: -P 2yn	Cell parameters from 17618 reflections
*a* = 7.0639 (7) Å	θ = 2.2–28.4°
*b* = 21.564 (2) Å	µ = 0.09 mm^−^^1^
*c* = 10.698 (1) Å	*T* = 173 K
β = 107.884 (2)°	Needle, orange
*V* = 1550.9 (3) Å^3^	0.16 × 0.09 × 0.06 mm
*Z* = 8	

### Data collection

**Table:**

Bruker Kappa DUO APEXII diffractometer	2379 reflections with *I* > 2σ(*I*)
Radiation source: fine-focus sealed tube	*R*_int_ = 0.055
graphite	θ_max_ = 28.4°, θ_min_ = 2.2°
0.5° φ scans and ω	*h* = −9→9
17618 measured reflections	*k* = −28→28
3887 independent reflections	*l* = −14→14

### Refinement

**Table:**

Refinement on *F*^2^	Primary atom site location: structure-invariant direct methods
Least-squares matrix: full	Secondary atom site location: difference Fourier map
*R*[*F*^2^ > 2σ(*F*^2^)] = 0.045	Hydrogen site location: inferred from neighbouring sites
*wR*(*F*^2^) = 0.117	H-atom parameters constrained
*S* = 1.00	*w* = 1/[σ^2^(*F*_o_^2^) + (0.0455*P*)^2^ + 0.3641*P*] where *P* = (*F*_o_^2^ + 2*F*_c_^2^)/3
3887 reflections	(Δ/σ)_max_ < 0.001
217 parameters	Δρ_max_ = 0.20 e Å^−^^3^
0 restraints	Δρ_min_ = −0.23 e Å^−^^3^

### Special details

**Table:**

Experimental. Half sphere of data collected using the Bruker *SAINT* software package. Crystal to detector distance = 45 mm; combination of φ and ω scans of 0.5°, 40 s per °, 2 iterations.
Geometry. All e.s.d.'s (except the e.s.d. in the dihedral angle between two l.s. planes) are estimated using the full covariance matrix. The cell e.s.d.'s are taken into account individually in the estimation of e.s.d.'s in distances, angles and torsion angles; correlations between e.s.d.'s in cell parameters are only used when they are defined by crystal symmetry. An approximate (isotropic) treatment of cell e.s.d.'s is used for estimating e.s.d.'s involving l.s. planes.
Refinement. Refinement of *F*^2^ against ALL reflections. The weighted *R*-factor *wR* and goodness of fit *S* are based on *F*^2^, conventional *R*-factors *R* are based on *F*, with *F* set to zero for negative *F*^2^. The threshold expression of *F*^2^ > σ(*F*^2^) is used only for calculating *R*-factors(gt) *etc*. and is not relevant to the choice of reflections for refinement. *R*-factors based on *F*^2^ are statistically about twice as large as those based on *F*, and *R*- factors based on ALL data will be even larger.

### Fractional atomic coordinates and isotropic or equivalent isotropic displacement parameters (Å^2^)

**Table:**

	*x*	*y*	*z*	*U*_iso_*/*U*_eq_	
O1A	0.2759 (2)	0.51665 (7)	0.83374 (13)	0.0500 (4)	
N1A	0.25666 (19)	0.46418 (6)	0.52163 (13)	0.0301 (3)	
C1A	0.2419 (2)	0.48240 (7)	0.39656 (16)	0.0283 (4)	
C2A	0.2385 (2)	0.43602 (8)	0.30235 (18)	0.0377 (4)	
H2A	0.2458	0.3935	0.3265	0.045*	
C3A	0.2247 (3)	0.45245 (10)	0.17677 (19)	0.0452 (5)	
H3A	0.2207	0.4211	0.1137	0.054*	
C4A	0.2162 (3)	0.51490 (10)	0.13944 (18)	0.0438 (5)	
H4A	0.2089	0.5254	0.0518	0.053*	
C5A	0.2184 (2)	0.56090 (9)	0.22770 (17)	0.0371 (4)	
H5A	0.2126	0.6031	0.2013	0.045*	
C6A	0.2292 (2)	0.54573 (7)	0.35861 (16)	0.0283 (4)	
C7A	0.2265 (2)	0.59065 (8)	0.45389 (16)	0.0313 (4)	
H7A	0.2169	0.6335	0.4319	0.038*	
C8A	0.2379 (2)	0.57216 (7)	0.57762 (17)	0.0319 (4)	
H8A	0.2340	0.6017	0.6427	0.038*	
C9A	0.2557 (2)	0.50841 (8)	0.60734 (16)	0.0294 (4)	
C10A	0.2734 (3)	0.48493 (9)	0.74045 (18)	0.0388 (4)	
H10A	0.2836	0.4413	0.7534	0.047*	
O1B	−0.60926 (19)	0.21792 (6)	0.27526 (14)	0.0513 (4)	
N1B	−0.1295 (2)	0.27109 (6)	0.43627 (14)	0.0332 (3)	
C1B	0.0608 (2)	0.25250 (7)	0.50026 (16)	0.0308 (4)	
C2B	0.2086 (3)	0.29846 (8)	0.54640 (18)	0.0388 (4)	
H2B	0.1752	0.3411	0.5320	0.047*	
C3B	0.4002 (3)	0.28133 (9)	0.61193 (18)	0.0431 (5)	
H3B	0.4990	0.3123	0.6430	0.052*	
C4B	0.4520 (3)	0.21879 (9)	0.63369 (18)	0.0417 (4)	
H4B	0.5860	0.2078	0.6786	0.050*	
C5B	0.3129 (2)	0.17325 (9)	0.59128 (17)	0.0381 (4)	
H5B	0.3501	0.1310	0.6076	0.046*	
C6B	0.1131 (2)	0.18895 (8)	0.52288 (16)	0.0307 (4)	
C7B	−0.0391 (2)	0.14417 (8)	0.47742 (17)	0.0341 (4)	
H7B	−0.0098	0.1013	0.4913	0.041*	
C8B	−0.2280 (2)	0.16321 (8)	0.41355 (17)	0.0343 (4)	
H8B	−0.3323	0.1339	0.3821	0.041*	
C9B	−0.2653 (2)	0.22733 (8)	0.39511 (16)	0.0311 (4)	
C10B	−0.4680 (3)	0.25053 (9)	0.32446 (18)	0.0408 (4)	
H10B	−0.4873	0.2941	0.3177	0.049*	

### Atomic displacement parameters (Å^2^)

**Table:**

	*U*^11^	*U*^22^	*U*^33^	*U*^12^	*U*^13^	*U*^23^
O1A	0.0461 (8)	0.0728 (10)	0.0322 (7)	−0.0039 (7)	0.0137 (6)	−0.0042 (7)
N1A	0.0271 (7)	0.0288 (7)	0.0334 (8)	−0.0013 (5)	0.0079 (6)	−0.0005 (6)
C1A	0.0212 (7)	0.0318 (9)	0.0314 (9)	−0.0018 (6)	0.0075 (6)	−0.0042 (7)
C2A	0.0332 (9)	0.0373 (10)	0.0425 (11)	−0.0025 (7)	0.0114 (8)	−0.0097 (8)
C3A	0.0361 (10)	0.0611 (13)	0.0397 (11)	−0.0047 (9)	0.0134 (8)	−0.0192 (9)
C4A	0.0320 (9)	0.0678 (14)	0.0311 (10)	−0.0047 (9)	0.0092 (7)	−0.0011 (9)
C5A	0.0290 (9)	0.0481 (11)	0.0340 (10)	−0.0020 (7)	0.0092 (7)	0.0042 (8)
C6A	0.0208 (7)	0.0331 (9)	0.0305 (9)	−0.0011 (6)	0.0073 (6)	0.0014 (7)
C7A	0.0295 (8)	0.0270 (8)	0.0373 (10)	−0.0005 (6)	0.0104 (7)	0.0017 (7)
C8A	0.0300 (8)	0.0306 (9)	0.0354 (10)	−0.0021 (7)	0.0105 (7)	−0.0063 (7)
C9A	0.0238 (8)	0.0341 (9)	0.0301 (9)	−0.0023 (6)	0.0079 (6)	−0.0011 (7)
C10A	0.0330 (9)	0.0471 (11)	0.0349 (10)	−0.0033 (8)	0.0085 (8)	0.0042 (8)
O1B	0.0357 (7)	0.0547 (9)	0.0539 (9)	0.0027 (6)	−0.0004 (6)	−0.0062 (7)
N1B	0.0370 (8)	0.0289 (7)	0.0336 (8)	0.0015 (6)	0.0107 (6)	−0.0016 (6)
C1B	0.0350 (9)	0.0304 (8)	0.0290 (9)	−0.0007 (7)	0.0129 (7)	−0.0026 (7)
C2B	0.0439 (10)	0.0333 (9)	0.0406 (10)	−0.0082 (8)	0.0148 (8)	−0.0047 (8)
C3B	0.0397 (10)	0.0475 (11)	0.0424 (11)	−0.0155 (8)	0.0131 (8)	−0.0071 (9)
C4B	0.0307 (9)	0.0530 (12)	0.0396 (10)	−0.0016 (8)	0.0081 (8)	−0.0013 (9)
C5B	0.0341 (9)	0.0399 (10)	0.0394 (10)	0.0017 (8)	0.0101 (8)	0.0023 (8)
C6B	0.0312 (8)	0.0321 (9)	0.0299 (9)	−0.0014 (7)	0.0110 (7)	−0.0001 (7)
C7B	0.0360 (9)	0.0257 (8)	0.0398 (10)	0.0009 (7)	0.0107 (8)	0.0010 (7)
C8B	0.0324 (9)	0.0306 (9)	0.0386 (10)	−0.0026 (7)	0.0091 (7)	−0.0036 (7)
C9B	0.0325 (8)	0.0310 (9)	0.0296 (9)	0.0025 (7)	0.0090 (7)	−0.0013 (7)
C10B	0.0404 (10)	0.0376 (10)	0.0413 (11)	0.0068 (8)	0.0080 (8)	0.0000 (8)

### Geometric parameters (Å, °)

**Table:**

O1A—C10A	1.206 (2)	O1B—C10B	1.202 (2)
N1A—C9A	1.325 (2)	N1B—C9B	1.321 (2)
N1A—C1A	1.367 (2)	N1B—C1B	1.368 (2)
C1A—C2A	1.415 (2)	C1B—C2B	1.414 (2)
C1A—C6A	1.420 (2)	C1B—C6B	1.420 (2)
C2A—C3A	1.364 (3)	C2B—C3B	1.370 (3)
C2A—H2A	0.9500	C2B—H2B	0.9500
C3A—C4A	1.401 (3)	C3B—C4B	1.398 (3)
C3A—H3A	0.9500	C3B—H3B	0.9500
C4A—C5A	1.366 (3)	C4B—C5B	1.364 (2)
C4A—H4A	0.9500	C4B—H4B	0.9500
C5A—C6A	1.417 (2)	C5B—C6B	1.417 (2)
C5A—H5A	0.9500	C5B—H5B	0.9500
C6A—C7A	1.410 (2)	C6B—C7B	1.416 (2)
C7A—C8A	1.361 (2)	C7B—C8B	1.362 (2)
C7A—H7A	0.9500	C7B—H7B	0.9500
C8A—C9A	1.408 (2)	C8B—C9B	1.410 (2)
C8A—H8A	0.9500	C8B—H8B	0.9500
C9A—C10A	1.480 (2)	C9B—C10B	1.485 (2)
C10A—H10A	0.9500	C10B—H10B	0.9500
			
C9A—N1A—C1A	117.09 (14)	C9B—N1B—C1B	117.33 (14)
N1A—C1A—C2A	118.25 (15)	N1B—C1B—C2B	118.42 (15)
N1A—C1A—C6A	122.37 (14)	N1B—C1B—C6B	122.15 (14)
C2A—C1A—C6A	119.38 (15)	C2B—C1B—C6B	119.42 (15)
C3A—C2A—C1A	119.90 (17)	C3B—C2B—C1B	119.80 (17)
C3A—C2A—H2A	120.1	C3B—C2B—H2B	120.1
C1A—C2A—H2A	120.1	C1B—C2B—H2B	120.1
C2A—C3A—C4A	120.97 (18)	C2B—C3B—C4B	120.84 (17)
C2A—C3A—H3A	119.5	C2B—C3B—H3B	119.6
C4A—C3A—H3A	119.5	C4B—C3B—H3B	119.6
C5A—C4A—C3A	120.70 (18)	C5B—C4B—C3B	120.90 (17)
C5A—C4A—H4A	119.7	C5B—C4B—H4B	119.5
C3A—C4A—H4A	119.7	C3B—C4B—H4B	119.5
C4A—C5A—C6A	120.07 (17)	C4B—C5B—C6B	120.08 (17)
C4A—C5A—H5A	120.0	C4B—C5B—H5B	120.0
C6A—C5A—H5A	120.0	C6B—C5B—H5B	120.0
C7A—C6A—C5A	123.16 (16)	C7B—C6B—C5B	123.05 (15)
C7A—C6A—C1A	117.88 (15)	C7B—C6B—C1B	117.98 (15)
C5A—C6A—C1A	118.96 (15)	C5B—C6B—C1B	118.96 (15)
C8A—C7A—C6A	119.46 (15)	C8B—C7B—C6B	119.36 (15)
C8A—C7A—H7A	120.3	C8B—C7B—H7B	120.3
C6A—C7A—H7A	120.3	C6B—C7B—H7B	120.3
C7A—C8A—C9A	118.70 (15)	C7B—C8B—C9B	118.56 (15)
C7A—C8A—H8A	120.6	C7B—C8B—H8B	120.7
C9A—C8A—H8A	120.6	C9B—C8B—H8B	120.7
N1A—C9A—C8A	124.46 (15)	N1B—C9B—C8B	124.62 (15)
N1A—C9A—C10A	113.76 (15)	N1B—C9B—C10B	114.65 (15)
C8A—C9A—C10A	121.78 (15)	C8B—C9B—C10B	120.73 (15)
O1A—C10A—C9A	125.30 (18)	O1B—C10B—C9B	124.52 (17)
O1A—C10A—H10A	117.4	O1B—C10B—H10B	117.7
C9A—C10A—H10A	117.4	C9B—C10B—H10B	117.7
			
C9A—N1A—C1A—C2A	−178.72 (14)	C9B—N1B—C1B—C2B	−179.40 (16)
C9A—N1A—C1A—C6A	1.0 (2)	C9B—N1B—C1B—C6B	−0.2 (2)
N1A—C1A—C2A—C3A	−179.74 (15)	N1B—C1B—C2B—C3B	179.39 (16)
C6A—C1A—C2A—C3A	0.5 (2)	C6B—C1B—C2B—C3B	0.2 (3)
C1A—C2A—C3A—C4A	0.8 (3)	C1B—C2B—C3B—C4B	0.2 (3)
C2A—C3A—C4A—C5A	−1.1 (3)	C2B—C3B—C4B—C5B	−0.6 (3)
C3A—C4A—C5A—C6A	0.0 (3)	C3B—C4B—C5B—C6B	0.7 (3)
C4A—C5A—C6A—C7A	−178.22 (16)	C4B—C5B—C6B—C7B	−179.28 (17)
C4A—C5A—C6A—C1A	1.3 (2)	C4B—C5B—C6B—C1B	−0.4 (3)
N1A—C1A—C6A—C7A	−1.7 (2)	N1B—C1B—C6B—C7B	−0.3 (2)
C2A—C1A—C6A—C7A	178.00 (15)	C2B—C1B—C6B—C7B	178.91 (16)
N1A—C1A—C6A—C5A	178.70 (14)	N1B—C1B—C6B—C5B	−179.26 (15)
C2A—C1A—C6A—C5A	−1.6 (2)	C2B—C1B—C6B—C5B	−0.1 (2)
C5A—C6A—C7A—C8A	−179.86 (15)	C5B—C6B—C7B—C8B	179.36 (17)
C1A—C6A—C7A—C8A	0.6 (2)	C1B—C6B—C7B—C8B	0.4 (2)
C6A—C7A—C8A—C9A	1.1 (2)	C6B—C7B—C8B—C9B	−0.1 (3)
C1A—N1A—C9A—C8A	0.9 (2)	C1B—N1B—C9B—C8B	0.6 (3)
C1A—N1A—C9A—C10A	−179.66 (13)	C1B—N1B—C9B—C10B	−179.01 (15)
C7A—C8A—C9A—N1A	−2.0 (2)	C7B—C8B—C9B—N1B	−0.4 (3)
C7A—C8A—C9A—C10A	178.60 (15)	C7B—C8B—C9B—C10B	179.14 (16)
N1A—C9A—C10A—O1A	179.95 (16)	N1B—C9B—C10B—O1B	176.94 (18)
C8A—C9A—C10A—O1A	−0.6 (3)	C8B—C9B—C10B—O1B	−2.6 (3)

### Hydrogen-bond geometry (Å, °)

**Table:**

*D*—H···*A*	*D*—H	H···*A*	*D*···*A*	*D*—H···*A*
C4A—H4A···O1A^i^	0.95	2.53	3.424 (2)	158

Symmetry codes: (i) *x*, *y*, *z*−1.
